# Prognostic value of postoperative albumin-bilirubin score in patients with acute type A aortic dissection after surgery: a propensity-score matched analysis

**DOI:** 10.1186/s12872-026-06058-3

**Published:** 2026-06-22

**Authors:** Jia-Wen Hu, Tao Shi

**Affiliations:** Department of Cardiovascular surgery, First Affiliated Hospital of Medical School, Xi’an Jiaotong University, No.277, Yanta West Road, Xi’an, 710061 P. R. China

**Keywords:** Acute type A aortic dissection, Postoperative ALBI, Prognostic value

## Abstract

**Background:**

Postoperative hepatic dysfunction is a frequent and severe complication in patients with acute type A aortic dissection (ATAAD) following emergency surgical repair, and it is strongly associated with adverse clinical outcomes and poor prognosis. The albumin-bilirubin (ALBI) score is an objective and well-validated indicator for evaluating hepatic function and reserve. Accumulating evidence has demonstrated its favorable prognostic value across a variety of clinical conditions, including cardiovascular diseases. Accordingly, this study was performed to comprehensively investigate the prognostic significance of the ALBI score in patients with ATAAD undergoing emergency surgical repair.

**Method:**

A total of 306 patients with ATAAD who underwent emergent repair surgery were enrolled in this single-center retrospective study. The primary outcomes of interest included delayed extubation, reintubation, neurological dysfunction, requirement for continuous renal replacement therapy (CRRT), and 30-day mortality. Propensity score matching (PSM) was performed to balance baseline characteristics between low and high postoperative ALBI (postALBI) score groups, followed by comparison of adverse outcome incidences. Multivariate logistic regression analysis was used to identify independent risk factors for poor prognosis.

**Results:**

After PSM, 191 matched pairs were obtained. The high postALBI group (≥-2.12) exhibited significantly higher rates of delayed extubation (40.34% vs. 18.48%, *P* < 0.001), CRRT requirement (31.93% vs. 10.92%, *P* < 0.01), and 30-day mortality (16.81% vs. 6.72%, *P* = 0.017) compared to the low postALBI group (<-2.12). Although the reintubation rate was higher in the high postALBI group (18.52% vs. 9.24%), the difference approached but did not reach statistical significance (*P* = 0.064). Multivariate analysis revealed that high postALBI was consistently identified as a common risk factor for all adverse outcomes, including delayed extubation (OR: 2.670; 95%CI: 1.080–6.601, *P* = 0.033), reintubation (OR: 3.071; 95%CI: 1.076–8.769, *P* = 0.036), neurological dysfunction (OR: 4.174; 95%CI: 1.354–12.868, *P* = 0.013), CRRT requirement (OR: 4.25; 95%CI: 1.544–11.699, *P* = 0.005), and 30-day mortality (OR: 3.215; 95%CI: 1.132–9.127, *P* = 0.028). The postALBI score exhibited superior discriminatory ability over individual liver function parameters (postoperative albumin and total bilirubin) for predicting multiple adverse clinical outcomes: its AUC and 95% CI were 0.668 (0.547–0.788, *P* = 0.006) for 30-day mortality, 0.737 (0.645–0.829, *P* < 0.001) for CRRT requirement, 0.730 (0.582–0.879, *P* = 0.002) for neurological dysfunction, 0.706 (0.588–0.824, *P* = 0.001) for reintubation, and 0.637 (0.548–0.725, *P* = 0.003) for delayed extubation.

**Conclusions:**

The postALBI score demonstrates independent prognostic potential for postoperative adverse events in patients with ATAAD undergoing emergent surgical repair. Owing to its simplicity and wide accessibility, this score may serve as a supportive tool for postoperative risk stratification and clinical decision-making in this high-risk population.

**Supplementary Information:**

The online version contains supplementary material available at https://doi.org/10.1186/s12872-026-06058-3.

## Introduction

Acute type A aortic dissection (ATAAD) is a life-threatening cardiovascular emergency characterized by rapid progression, high morbidity, and substantial mortality [1, 2]. Despite remarkable advances in surgical techniques and perioperative management, the prognosis of patients with ATAAD remains unsatisfactory. Previous studies have reported in-hospital mortality rates as high as 30%, with 1-year and 5-year post-discharge mortality ranging from 4% to 48% and 9%-63%, respectively [2]. Therefore, accurate risk stratification and early identification of high-risk patients are critical to optimize therapeutic strategies and improve clinical outcomes.

Postoperative hepatic dysfunction is a frequent and severe complication in patients with ATAAD following emergency surgical repair, and it is strongly associated with adverse clinical outcomes and poor prognosis [3–5]. Emerging evidence indicates that perioperative hepatic impairment is closely linked to elevated in-hospital and 30-day mortality, as well as increased risks of multiple organ dysfunction and prolonged hospitalization, further highlighting the importance of reliable liver function assessment for risk stratification [3–5]. The albumin-bilirubin (ALBI) score, derived from serum albumin and total bilirubin, is a simple, objective, and reproducible index for evaluating liver function and reserve capacity. In contrast to traditional systems such as the Child-Pugh grade and Model for End-Stage Liver Disease (MELD) score, the ALBI score is independent of subjective clinical parameters, supporting its broad clinical utility [6].

In recent years, the prognostic value of the ALBI score has been extensively validated across a spectrum of clinical disorders, ranging from chronic liver disease to acute and chronic cardiovascular conditions [7–10]. In patients with acute heart failure, the ALBI score at discharge has been established as a useful tool for risk stratification with respect to all-cause mortality and heart failure rehospitalization [8]. Similarly, in patients undergoing transcatheter aortic valve implantation (TAVI), both in-hospital mortality and long-term mortality rates increased progressively across ALBI tertiles, and the preprocedural ALBI score exhibited a near-linear association with mortality risk [7]. A recent retrospective study further confirmed that the preoperative ALBI score was independently associated with in-hospital and long-term mortality in patients with type B aortic dissection [11]. However, the prognostic value of the ALBI score-especially the postoperative ALBI (postALBI)score-in patients with ATAAD undergoing emergent surgical repair remains unclear.

Existing evidence indicates that postoperative hypoalbuminemia and hyperbilirubinemia are separately associated with amplified inflammatory responses and increased early and long-term mortality in aortic dissection patients [12, 13]. Since the ALBI score integrates both albumin and bilirubin levels, it may provide a more comprehensive evaluation of liver function and systemic status than individual biomarkers alone. Accordingly, the present study investigated the association between the postALBI score and adverse clinical outcomes in patients with ATAAD undergoing emergency surgery, and to evaluate its potential as a practical prognostic stratification tool.

## Materials and methods

### Study subjects

**Fig. 1:**
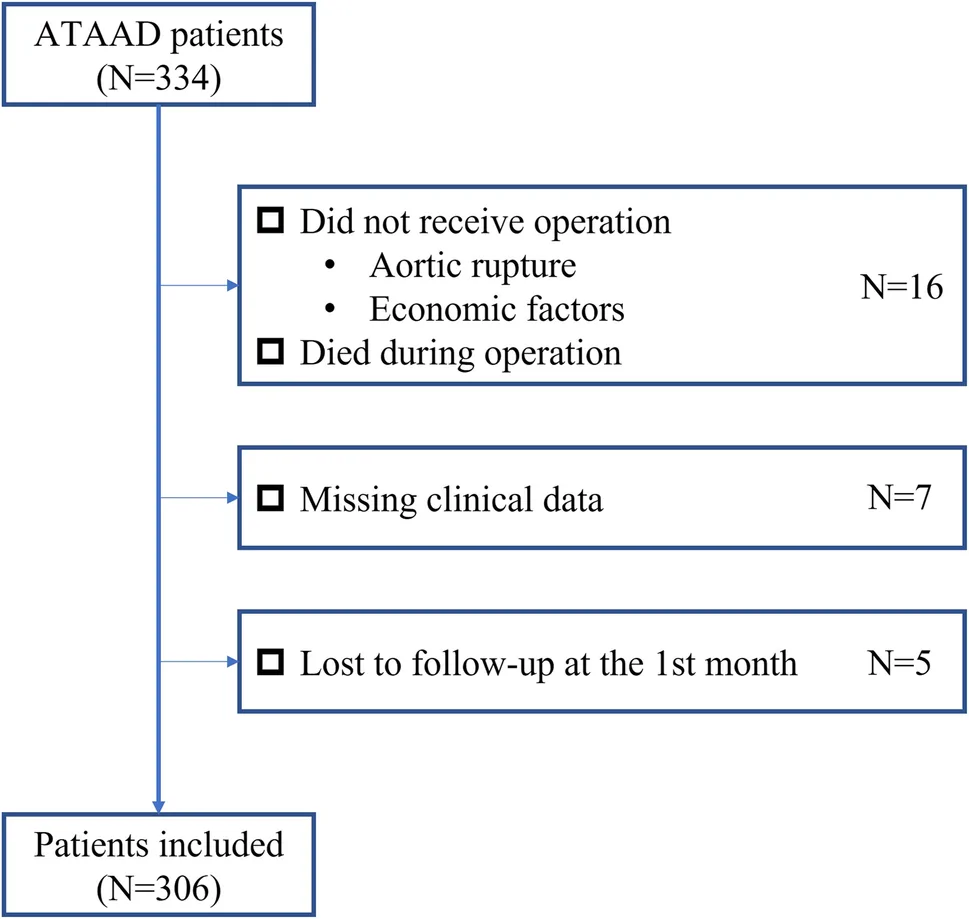
Flow chart of screening

This single-center retrospective study was conducted at the First Affiliated Hospital of Xi’an Jiaotong University. We consecutively reviewed 334 patients aged > 18 years who were admitted with a primary diagnosis of ATAAD between September 2020 and March 2022. The diagnosis of ATAAD was confirmed by computed tomographic angiography (CTA). Patients were excluded if they did not undergo surgical therapy due to aortic rupture, economic constraints, or died during surgery (*n* = 16), had missing clinical data (*n* = 7), or were lost to follow-up at 1 month postoperatively (*n* = 5). A total of 306 patients were ultimately included in the final analysis (Fig. 1). This study was conducted in accordance with the latest version of the Declaration of Helsinki and was approved by the Medical Science Research Ethics Committee of the First Affiliated Hospital of Xi’an Jiaotong University (Approval No.: 2022 − 578). Written informed consent was waived due to the retrospective nature of the study.

### Data collection

Perioperative clinical data, including demographic characteristics, laboratory parameters, and surgical information, were retrospectively retrieved from electronic medical records. Postoperative parameters were all measured within 24 h after surgery. Body mass index (BMI) was calculated as weight (kg) divided by height squared (m²). The ALBI score was computed using the formula: 0.66×log10(total bilirubin [TBil], µmol/L) – 0.085×albumin (g/L) [6]. The primary outcomes evaluated were delayed extubation (defined as mechanical ventilation duration > 48 h), reintubation, neurological dysfunction, requirement for continuous renal replacement therapy (CRRT), and 30-day mortality. Neurological dysfunction was defined as follows: (1) Acute ischemic stroke and intracerebral hemorrhage, diagnosed by postoperative neurological evaluation combined with head CT, computed tomography angiography (CTA), and/or computed tomography perfusion (CTP); (2) Clinical paraplegia confirmed by standardized neurological examination; (3) Hypoxic-ischemic encephalopathy presenting as coma, diagnosed based on neurological examination and typical cranial CT findings, including bilateral basal ganglia hypodensities, diffuse cerebral edema with effacement of cerebral sulci, loss of normal gray-white matter differentiation, and bilateral watershed hypodensities. All patients were followed up at 1 month postoperatively through outpatient re-examination or telephone consultation.

Surgical procedures were performed by a single experienced surgical team under general anesthesia. Cardiopulmonary bypass (CPB) was established at various sites (right axillary artery, femoral artery, innominate artery, or double arterial cannulation) based on individual patient conditions. Patients were cooled to a nasopharyngeal temperature of 26–28 °C. The ascending aorta was clamped, and cold blood cardioplegia was infused through the coronary ostia to achieve cardiac arrest. Antegrade cerebral perfusion was established via axillary perfusion with a clamped brachiocephalic artery and direct cannulation of the left common carotid and subclavian arteries for brain protection. Detailed surgical procedures were tailored to the specific pathological changes of each patient (Table 1).

**Table 1:** Surgical types of included patients with ATAAD

Surgical procedure	Number of patients (%)
Ascending aorta replacement + Sun’s procedure	199 (65.03%)
Ascending aorta replacement + Sun’s procedure + Ascending aorto-femoral artery bypass	14 (4.57%)
Ascending aorta replacement + Sun’s procedure + AVP	14 (4.57%)
Ascending aorta replacement + Sun’s procedure + Ascending aorto-femoral artery bypass + CABG	3 (0.98%)
Ascending aorta replacement + Sun’s procedure + endovascular stenting	9 (2.94%)
Ascending aorta replacement+ hemiarch replacement	2 (0.65%)
Bentall	4 (1.31%)
Bentall+ hemiarch replacement	5 (1.63%)
Bentall+ hemiarch replacement + MVP	1 (0.33%)
Bentall + CABG	1 (0.33%)
Bentall + MVR + TVP	1 (0.33%)
Bentall + Sun’s procedure	36 (11.76)
Bentall + Sun’s procedure + CABG	3 (0.98%)
Bentall + Sun’s procedure + Ascending aorto-femoral artery bypass	3 (0.98%)
Bentall + Sun’s procedure + endovascular stenting	1(0.33%)
David + Sun’s procedure	5 (1.63%)
David + Sun’s procedure+ CABG	2 (0.65%)
David + Sun’s procedure + Ascending aorto-femoral artery bypass + CABG	1 (0.33%)
David + Sun’s procedure+ endovascular stenting	1 (0.33%)
Wheat + Sun’s procedure	1 (0.33%)

Sun’s procedure = total arch replacement and stented elephant trunk implantation, *AVP* aortic valve plasty, *MVP *mitral valve plasty, *MVR* mitral valve replacement, *CABG* coronary artery bypass grafting, *TVP *tricuspid valve plasty

### Statistical analysis

Statistical analyses were performed using IBM SPSS Statistics 24.0 (IBM Corp., Chicago, IL, USA). The Kolmogorov–Smirnov test was used to assess the distribution of continuous variables. Normally distributed variables are presented as mean ± standard deviation (SD), while skewed variables are reported as median with interquartile range (IQR). Categorical variables are summarized as counts and percentages. Between-group comparisons were conducted using Student’s t-test or the Mann–Whitney U test for continuous variables, and the χ² test or Fisher’s exact test for categorical variables, as appropriate.

For stratification by the postoperative ALBI score, the median value of the ALBI scores in the entire cohort was adopted as the cut-off point. Patients were accordingly divided into a low ALBI group ( < − 2.12) and a high ALBI group ( ≥ − 2.12). This grouping strategy was chosen to ensure relatively balanced sample sizes between the two subgroups, which is essential for subsequent propensity score matching (PSM) to balance baseline characteristics and maintain adequate statistical power for the analysis of multiple adverse clinical outcomes.

Univariate and multivariate logistic regression analyses were performed to identify risk factors for adverse outcomes. Variables with *P* < 0.05 in univariate analysis, together with clinically relevant baseline characteristics, were entered into a backward stepwise multivariate model to identify independent risk factors for each outcome.

To minimize potential confounding and selection bias, 1:1 propensity score matching (PSM) was performed using the nearest-neighbor matching method with a caliper width set at 0.1 times the standard deviation of the logit of the propensity score. Variables included in the propensity score model were age, gender, BMI, hypertension, and smoking history. Receiver operating characteristic (ROC) curve analysis was conducted to evaluate the predictive performance of the ALBI score for adverse clinical outcomes. Calibration was assessed using the Hosmer–Lemeshow test.

All statistical tests were two-tailed, and a P-value < 0.05 was considered statistically significant. Bonferroni correction was applied to adjust for multiple comparisons, with the significance threshold maintained at *P* < 0.05 after correction. Sensitivity analyses based on multiple imputation for missing data are presented in the Supplemental Table. Standardized mean differences (SMD) of baseline covariates before and after PSM are visualized in the Love plot provided in the Supplemental Figure.

## Results

### Baseline characteristics

The study cohort consisted of 306 ATAAD patients who underwent surgical repair, including 230 males (75.2%) and 76 females (24.8%) with a median age of 53.5 [45.3, 62.0] years. Hypertension was present in 48.4% of patients. The overall incidences of delayed extubation, reintubation, neurological dysfunction, CRRT requirement, and 30-day mortality were 33.66%, 13.40%, 9.80%, 21.24%, and 15.03%, respectively. Detailed surgical procedures and case numbers are presented in Table 1.

Patients were divided into the low postALBI score group (< -2.12) and high postALBI score group (≥ -2.12) based on the median postALBI score of the entire study cohort. After PSM, 119 matched pairs were obtained. Compared to the low postALBI group, the high postALBI group had a longer surgical duration. Preoperatively, the high postALBI group exhibited higher levels of white blood cell count (WBC), blood urea nitrogen (BUN), creatinine (CRE), D-dimer (DD), and fibrin degradation product (FDP), as well as lower levels of platelet count (PLT) and albumin. Postoperatively, the high postALBI group also had lower levels of PLT, albumin, and fibrinogen (FIB), and higher levels of CRE. No significant differences were observed in other baseline characteristics between the two groups (Table 2).

**Table 2:** Baseline characteristics for patients before and after propensity matching stratified by postoperative ALBI

Clinical parameters	Unmatched dataset			Matched (1:1) dataset		
	Low postALBI (*N* = 154)	High postALBI (*N* = 152)	*P* value	Low postALBI (*N* = 119)	High postALBI (*N* = 119)	*P* value
Age, yrs	52.10 ± 12.28	54.59 ± 11.14	0.066	52.60 ± 11.76	52.90 ± 11.05	0.838
Male, n(%)	109 (70.78)	121 (79.61)	0.074	92 (77.3)	92 (77.3)	1.000
BMI, kg/m2	25.83 ± 4.11	25.58 ± 3.89	0.606	25.57 ± 4.14	25.54 ± 3.86	0.949
Hypertension, %	46.75	50.00	0.570	49.58	49.58	1.000
Smoking, %	33.77	50.00	**0.004**	40.33	46.22	0.359
CPB time, min	153.43 ± 36.50	163.63 ± 41.31	**0.028**	151.94 ± 36.74	160.59 ± 38.80	0.087
ACC time, min	87.98 ± 22.63	91.65 ± 26.31	0.205	86.51 ± 20.91	90.69 ± 25.43	0.180
MHCA, min	19.95 ± 4.68	20.46 ± 5.05	0.401	19.78 ± 4.61	20.45 ± 5.38	0.338
Blood transfusion, ml	1122 ± 446	1297 ± 678	**0.012**	1111 ± 449	1248 ± 618	0.063
Preoperative biochemical data						
WBC, 10^9/L	10.88 ± 3.72	11.83 ± 4.77	0.068	10.90 ± 3.84	12.29 ± 4.82	**0.019**
Hb, g/L	130.11 ± 23.27	132.77 ± 22.79	0.339	132.22 ± 23.65	133.65 ± 22.72	0.648
PLT, 10^9/L	172.45 ± 59.07	144.96 ± 66.07	**< 0.001**	174.47 ± 56.93	147.81 ± 65.63	**0.002**
ALT, U/L	30.00[18.00, 41.00]	28.00[18.00, 40.50]	0.206	28.00[18.00, 40.50]	28.00[17.00, 41.00]	0.221
AST, U/L	24.00[20.00, 31.00]	25.00[19.00, 50.25]	0.171	24.00[19.50, 32.00]	25.50[19.00, 52.75]	0.202
ALB, g/L	36.05 ± 4.86	34.60 ± 4.42	**0.011**	36.33 ± 4.71	34.63 ± 4.54	**0.008**
TBIL, umol/L	15.90[9.80, 24.35]	17.5[14.80, 22.90]	0.384	16.40[12.40, 22.90]	18.08[14.88, 24.30]	0.521
BUN, mmol/L	7.55 ± 3.96	8.52 ± 4.34	0.054	7.31 ± 2.93	8.40 ± 4.18	**0.029**
CRE, umol/L	74.00[58.75, 100.25]	78.50[58.00, 135.50]	0.073	76.00[62.00, 101.5]	76.00[57.75, 136.00]	0.052
FIB, g/L	2.81 ± 1.46	2.73 ± 1.49	0.677	2.79 ± 1.40	2.81 ± 1.48	0.888
DD, mg/L	7.67[2.16, 18.72]	13.85[3.89, 39.45]	**0.008**	7.33[1.91, 17.35]	14.60[5.08, 35.54]	**0.002**
FDP, mg/L	23.03[7.89, 50.26]	41.07[12.20,111.12]	**0.028**	21.42[6.05, 46.66]	45.74[12.61, 96.14]	**0.006**
IL-6, pg/mL	46.50[20.25, 100.25]	58.96[27.26, 128.43]	0.014	42.80[19.59, 57.88]	46.39[ 25.39, 126.90]	0.030
Postoperative laboratory parameters						
WBC, 10^9/L	11.54 ± 4.07	11.11 ± 3.78	0.346	11.04 ± 3.91	11.42 ± 3.72	0.437
Hb, g/L	105.92 ± 14.43	104.97 ± 14.82	0.581	105.41 ± 14.94	106.24 ± 15.00	0.669
PLT, 10^9/L	86.47 ± 39.38	71.22 ± 37.81	**< 0.001**	86.41 ± 37.07	72.13 ± 39.47	**0.004**
ALT, U/L	30.00[20.00, 43.00]	32.00[23.00, 58.75]	0.319	30.00[20.00, 43.00]	31.00[24.00, 59.00]	0.181
AST, U/L	55.00[42.25, 82.50]	71.00[47.00, 145.00]	0.247	54.00[42.00, 83.00]	70.00[48.00, 140.00]	0.177
ALB, g/L	40.48 ± 3.04	33.55 ± 3.94	**< 0.001**	40.51 ± 3.05	33.89 ± 3.79	**< 0.001**
TBIL, umol/L	35.70[27.08, 51.38]	42.50[32.50, 57.45]	0.188	37.50[28.10, 52.20]	43.50[34.50, 60.80]	0.195
BUN, mmol/L	11.76 ± 4.84	13.29 ± 5.46	0.013	11.57 ± 4.65	17.95 ± 48.85	0.158
CRE, umol/L	111.00[86.00,145.25]	142.00[102.50,207.50]	**0.010**	113.00[87.00,135.00]	144.00[95.00, 222.00]	**0.003**
FIB, g/L	3.11 ± 0.84	2.80 ± 0.91	**0.004**	3.06 ± 0.81	2.81 ± 0.88	**0.029**
DD, mg/L	14.65[7.29, 20.36]	17.82[11.88, 26.57]	0.499	14.84[7.30, 20.74]	17.82[12.00, 26.89]	0.866
FDP, mg/L	47.20[23.16, 73.49]	60.98[37.98, 92.91]	0.161	47.65[24.26, 73.76]	61.51[37.53, 93.88]	0.432
IL-6, pg/mL	116.00[71.19, 190.40]	131.70[82.59, 312.20]	0.058	115.00[72.15, 190.40]	125.40[94.55, 307.18]	0.081

Bold values represent statistically significant differences

*PostALBI *Postoperative Albumin-bilirubin score, *BMI *Body mass index, *CPB* Cardiopulmonary bypass, *ACC* Aortic cross-clamp, *MHCA* Mild hypothermic circulatory arrest, *WBC *White blood cell, *Hb* haemoglobin, *PLT* Platelet, *ALT* Alanine aminotransferase, *AST* Glutamate aminotransferase, *TBIL* Total bilirubin, *BUN *Blood urea nitrogen, *CRE* Creatinine, *FIB *Fibrinogen, *DD* D dimer, *FDP* Fibrin degradation products

Statistical significance was defined as *P* < 0.05

### Comparison of adverse clinical events between two groups

**Fig. 2:**
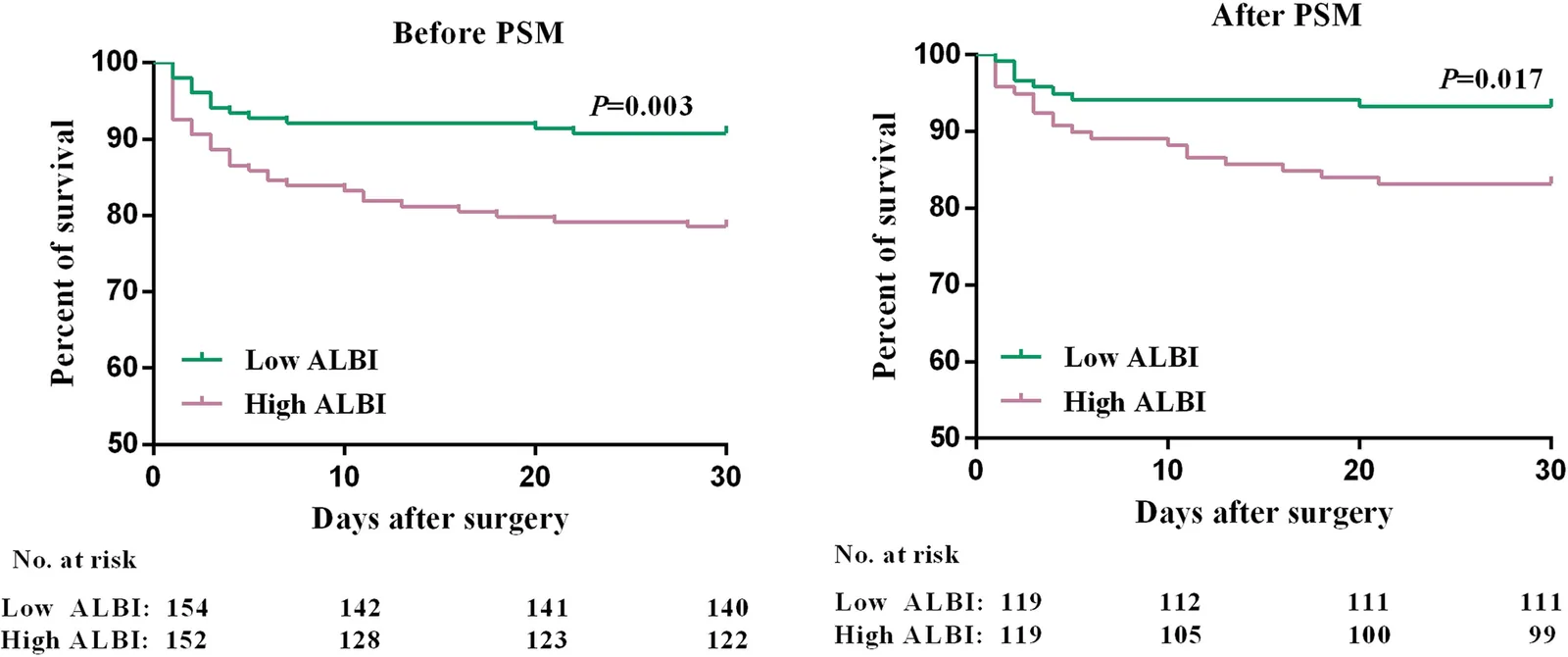
K-M curves of 30d mortality before and after PSM

As shown in Table 3; Fig. 2, after PSM, the high postALBI group had a significantly higher delayed extubation rate compared to the low postALBI group (40.34% vs. 18.48%, *P* < 0.001). Although the reintubation rate was higher in the high postALBI group (18.52% vs. 9.24%), the difference did not reach statistical significance (*P* = 0.064). The incidence of neurological dysfunction was 13.44% in the high postALBI group and 6.72% in the low postALBI group, with no significant between-group difference. The CRRT requirement rate (31.93% vs. 10.92%, *P* < 0.01) and 30-day mortality rate (16.81% vs. 6.72%, *P* = 0.017) were significantly higher in the high postALBI group.

**Table 3:** Postoperative characteristics of the two groups

Clinical parameters	Unmatched dataset			Matched (1:1) dataset		
	Low postALBI (*N* = 154)	High postALBI (*N* = 152)	*P* value	Low postALBI (*N* = 119)	High postALBI (*N* = 119)	*P* value
Delayed extubation, %	24.03	43.42	**< 0.001**	18.48	40.34	**< 0.001**
Reintubation, %	9.09	17.76	**0.038**	9.24	18.52	0.064
Neurological dysfunction, %	7.79	11.84	0.234	6.72	13.44	0.085
CRRT, %	13.63	28.95	**< 0.001**	10.92	31.93	**< 0.001**
30d mortality, %	9.09	21.05	**0.003**	6.72	16.81	**0.017**

Bold values represent statistically significant differences

*PostALBI *Postoperative Albumin-bilirubin score, *CRRT* Continuous renal replacement therapy

Statistical significance was defined as *P* < 0.05

### Univariate and multivariate logistic regression analysis

Univariate and multivariate logistic regression analyses were performed to identify independent risk factors for adverse clinical outcomes in the PSM cohort (Table 4). For delayed extubation, both preALBI (OR: 3.338; 95%CI: 1.317–8.455, *P* = 0.011) and postALBI (OR: 2.670; 95%CI: 1.080–6.601, *P* = 0.033) were independent predictive factors. Risk factors for reintubation included low preoperative hematocrit (preHCT) (OR: 0.918; 95%CI: 0.846–0.996, *P* = 0.040), low postoperative FIB (postFIB) (OR: 0.407; 95%CI: 0.190–0.869, *P* = 0.020), and high postALBI (OR: 3.071; 95%CI: 1.076–8.769, *P* = 0.036). Longer CPB time (OR: 1.020; 95%CI: 1.010–1.031, *P* < 0.01) and high postALBI (OR: 4.174; 95%CI: 1.354–12.868, *P* = 0.013) were independent predictors of neurological dysfunction. For CRRT requirement, high preoperative WBC (preWBC) (OR: 1.616; 95%CI: 1.049–1.285, *P* = 0.004), low postoperative PLT (postPLT) (OR: 0.969; 95%CI: 0.950–0.988, *P* = 0.002), and high postALBI (OR: 4.25; 95%CI: 1.544–11.699, *P* = 0.005) were significant risk factors. Low postFIB (OR: 0.317; 95%CI: 0.163–0.615, *P* < 0.001) and high postALBI (OR: 3.215; 95%CI: 1.132–9.127, *P* = 0.028) were independently associated with 30-day mortality. Notably, high postALBI was a common risk factor for all adverse outcomes evaluated in this study.

**Table 4:** Univariate and multivariate regression analysis for poor clinical outcomes

Clinical parameters	Unmatched dataset				Matched (1:1) dataset			
	Univariate analysis	*P* value	Multivariate analysis	*P* value	Univariate analysis	*P* value	Multivariate analysis	*P* value
Delayed extubation								
CPB	1.015[1.007,1.024]	**< 0.001**	1.014[1.005,1.024]	**0.003**	1.013[1.004,1.021]	**0.003**		
Length of surgery	1.347[1.113,1.630]	**0.002**			1.356[1.112,1.653]	**0.003**		
preWBC	1.150[1.046,1.264]	**0.004**						
preALBI	2.492[1.178,5.274]	**0.017**	2.296[1.013,5.204]	**0.047**	2.897[1.275,6.584]	**0.011**	3.338[1.317,8.455]	**0.011**
postALBI	2.136[1.029,4.434]	**0.042**			2.808[1.251,6.302]	**0.012**	2.670[1.080,6.601]	**0.033**
Reintubation								
age	1.052[1.014,1.092]	**0.007**	1.046[1.001,1.092]	**0.045**	1.038[1.004,1.074]	**0.029**		
CPB					1.012[1.002,1.022]	**0.018**		
Length of surgery					1.441[1.142,1.819]	**0.002**		
preHCT					0.942[0.890,0.998]	**0.044**	0.918[0.846,0.996]	**0.040**
postPLT	0.983[0.968,0.999]	**0.035**			0.978[0.964,0.992]	**0.003**		
postFIB	0.341[0.178,0.655]	**0.001**	0.413[0.204,0.834]	**0.014**	0.317[0.169,0.596]	**< 0.001**	0.407[0.190,0.869]	**0.020**
postALBI	9.588[2.812,32.693]	**< 0.001**	8.197[2.317,29.000]	**0.001**	3.806[1.401,10.334]	**0.009**	3.071[1.076,8.769]	**0.036**
Neurological dysfunction								
CPB	1.017[1.008,1.025]	**< 0.001**			1.022[1.011,1.032]	**< 0.001**	1.020[1.010,1.031]	**< 0.001**
Length of surgery	1.498[1.203,1.864]	**< 0.001**	1.387[1.029,1.871]	**0.032**	1.719[1.305,2.265]	**< 0.001**		
prePLT	0.991[0.982,0.999]	**0.027**			0.989[0.979,0.999]	**0.035**		
postPLT	0.978[0.964,0.991]	**0.001**			0.974[0.958,0.989]	**0.001**		
postALBI	2.785[1.137,6.818]	**0.025**			5.276[1.820,15.296]	**0.002**	4.174[1.354,12.868]	**0.013**
CRRT								
Length of surgery					1.472[1.190,1.821]	**< 0.001**		
prePLT	0.989[0.982,0.996]	**0.003**			0.983[0.975,0.990]	**< 0.001**		
preWBC	1.161[1.062,1.270]	**0.001**	1.137[1.033,1.252]	**0.009**	1.191[1.093,1.297]	**< 0.001**	1.161[1.049,1.285]	**0.004**
postPLT	0.979[0.968,0.991]	**< 0.001**			0.964[0.951,0.977]	**< 0.001**	0.969[0.950,0.988]	**0.002**
postFIB	0.457[0.289,0.723]	**< 0.001**	0.502[0.293,0.862]	**0.012**	0.368[0.226,0.597]	**< 0.001**		
postALBI	6.108[2.497,14.94]	**< 0.001**	4.369[1.566,12.189]	**0.005**	7.829[3.261,18,792]	**< 0.001**	4.250[1.544,11.699]	**0.005**
30-day mortality								
CPB	1.011[1.004,1.019]	**0.004**						
Length of surgery	1.579[1.304,1.913]	**< 0.001**	1.313[1.001,1.722]	**0.049**	1.416[1.122,1.786]	**0.003**		
postWBC	1.120[1.027,1.220]	**0.010**	1.123[1.022,1.235]	**0.016**	1.107[1.003,1.222]	**0.043**		
postFIB					0.274[0.141,0.531]	**< 0.001**	0.317[0.163,0.615]	**< 0.001**
postALBI	4.080[1.755,9.484]	**0.001**	3.760[1.482,9.538]	**0.005**	4.443[1.644,12.008]	**0.003**	3.215[1.132,9.127]	**0.028**

Bold values represent statistically significant differences

*Pre *preoperative, *Post *postoperative, *CPB* Cardiopulmonary bypass, *CRRT* continuous renal replacement therapy, *WBC *White blood cell, *ALBI* Albumin-bilirubin score, *HCT* Hematocrit, *PLT* Platelet, *FIB *Fibrinogen

Statistical significance was defined as *P* < 0.05

### Comparison of postALBI, postALB, and postTBIL in predicting adverse prognosis

**Fig. 3:**
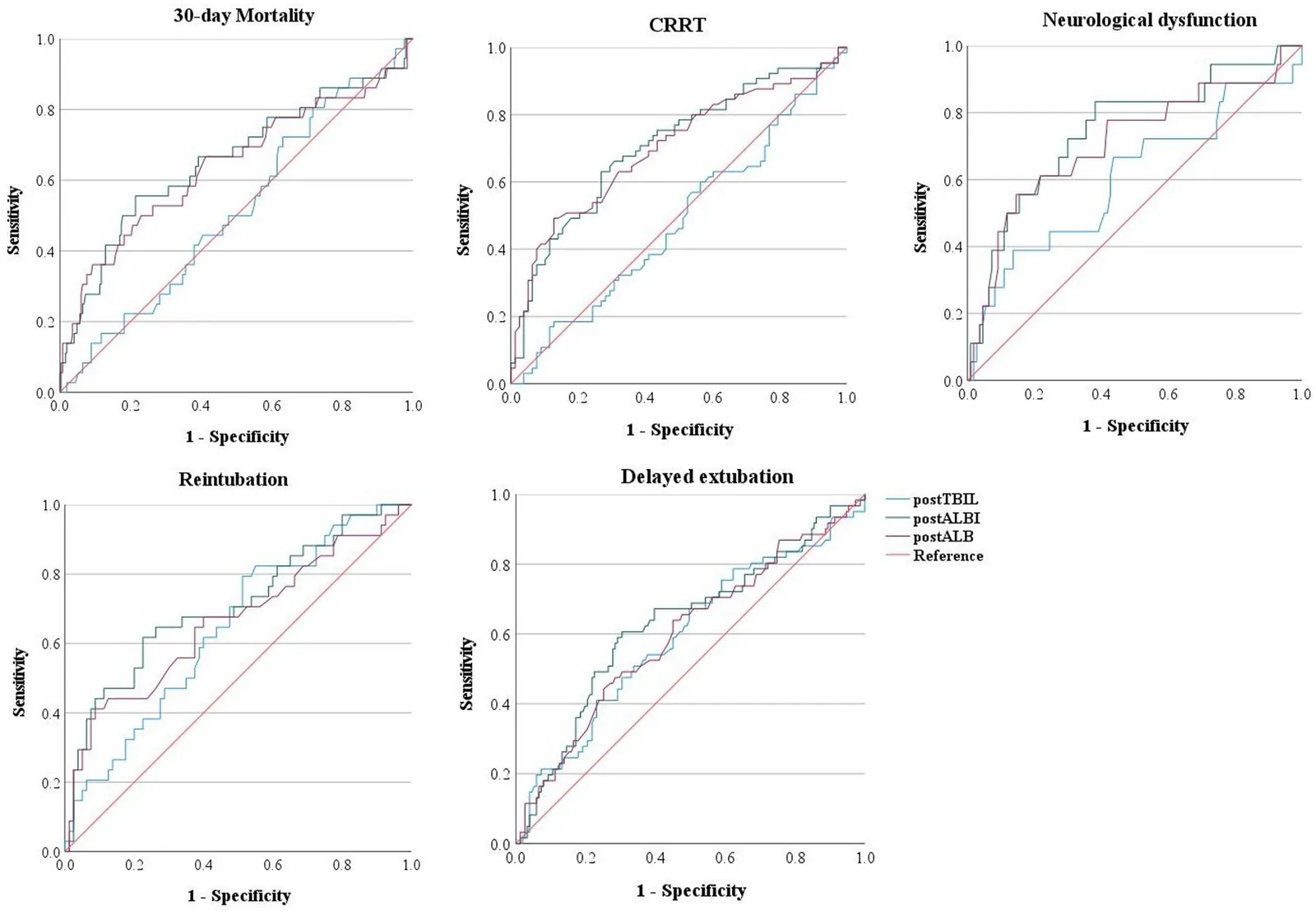
Receiver operating characteristic curves of postoperative ALBI, ALB, and TBIL in predicting adverse clinical outcomes

ROC curve analysis was performed to assess the predictive value of postALBI, postoperative albumin (postALB), and postoperative total bilirubin (postTBIL) for postoperative adverse outcomes (Table 5; Fig. 3). For 30-day mortality, the AUC of postALBI was 0.668 (95% CI: 0.547–0.788, *P* = 0.006), which was higher than postALB (AUC = 0.651, 95% CI: 0.528–0.773, *P* = 0.016). PostALBI also showed superior predictive performance for CRRT requirement (AUC = 0.737, 95% CI: 0.645–0.829, *P* < 0.001), neurological dysfunction (AUC = 0.730, 95% CI: 0.582–0.879, *P* = 0.002), reintubation (AUC = 0.706, 95% CI: 0.588–0.824, *P* = 0.001), and delayed extubation (AUC = 0.637, 95% CI: 0.548–0.725, *P* = 0.003) compared with postALB and postTBIL. Overall, postALBI exhibited a better discriminatory capacity than individual liver function parameters in predicting adverse clinical outcomes.

**Table 5:** Comparison of postALBI, postALB, and postTBIL in predicting adverse prognosis

Outcomes and Variables	AUC (95% CI)	*P* value	Sensitivity,%	Sepcificity,%
30-day mortality				
postALBI	0.668 (0.547–0.788)	**0.006**	67.9	62.1
postALB	0.651 (0.528–0.773)	**0.016**	67.9	60.7
CRRT				
postALBI	0.737 (0.645–0.829)	**< 0.001**	74.5	65.6
postALB	0.729 (0.633–0.824)	**< 0.001**	66.7	67.2
Neurological dysfunction				
postALBI	0.730 (0.582–0.879)	**0.002**	81.3	59.1
postALB	0.698 (0.538–0.858)	**0.015**	50.0	86.4
Reintubation				
postALBI	0.706 (0.588–0.824)	**0.001**	61.3	75.4
postTBIL	0.649 (0.536–0.763)	**0.010**	80.6	44.6
postALB	0.655 (0.530–0.780)	**0.015**	64.5	63.1
Delayed extubation				
postALBI	0.637 (0.548–0.725)	**0.003**	67.2	63.1
postALB	0.607 (0.518–0.697)	**0.019**	63.8	57.4

Bold values represent statistically significant differences

*postALBI *postoperative Albumin-bilirubin score, *postALB *postoperative albumin, *postTBIL *postoperative total bilirubin, *AUC* area under the curve, *CI *confidence interval, *CRRT* continuous renal replacement therapy

Statistical significance was defined as *P* < 0.05

## Discussion

This study comprehensively evaluated the prognostic value of the postALBI score in ATAAD patients undergoing emergent surgical repair. The key finding was that an elevated postALBI score served as an independent predictor of multiple adverse clinical outcomes, including delayed extubation, reintubation, requirement for CRRT, neurological dysfunction, and 30-day mortality. Furthermore, the postALBI score exhibited better or at least equivalent predictive performance for short-term adverse outcomes compared with postALB and postTBIL alone. These results highlight the potential of the postALBI score as a valuable tool for risk stratification and prognostic assessment in this high-risk patient population.

The overall incidences of CRRT requirement (21.24%), neurological dysfunction (9.80%), and 30-day mortality (15.03%) in our study are consistent with those reported in previous multicenter studies (15%-22%, 10%-17%, and 14%-22%, respectively), indicating the generalizability of our findings [3, 4, 14]. The ALBI score, originally developed for assessing liver function and long-term prognosis in patients with liver diseases and cancer [6], has recently been shown to have prognostic value in various cardiovascular diseases, such as valvular heart disease, heart failure, cardiomyopathy, atrial septal defect, and type B aortic dissection [5, 9, 11, 15, 16]. However, this is the first study to systematically investigate the association between the postALBI score and postoperative outcomes in ATAAD patients.

The underlying mechanisms linking high postALBI to adverse outcomes in ATAAD patients are complex and may involve multiple pathways. First, the onset of aortic dissection triggers a profound systemic inflammatory response, which is further amplified by cardiopulmonary bypass (CPB) and aortic cross-clamping during surgery. Such excessive inflammation increases capillary permeability, leading to albumin extravasation and subsequent hypoalbuminemia [17]. Serum albumin exerts critical anti-inflammatory, antioxidant, and free-radical scavenging functions, with approximately three-quarters of serum free-radical trapping activity attributed to albumin [18]. Hypoalbuminemia is associated with sustained inflammation, dysregulated coagulation and fibrinolysis, impaired immune function, and increased vulnerability to infections and thrombotic events, all of which contribute to unfavorable clinical outcomes [19–21].

Second, hyperbilirubinemia, another key component of the ALBI score, is mainly induced by CPB-related hypoperfusion, exaggerated inflammation, and hemolysis [12, 22]. Although unconjugated bilirubin possesses intrinsic anti-inflammatory and antioxidant properties, postoperative hyperbilirubinemia generally reflects the severity of systemic inflammation and oxidative stress [22]. A meta-analysis indicated that postoperative hyperbilirubinemia is associated with prolonged ICU and hospital stays, increased requirements for invasive ventilation and new-onset dialysis, and higher mortality in patients undergoing cardiac surgery with CPB [23]. Additionally, perioperative hypoxia is common in ATAAD patients, with preoperative and postoperative hypoxia rates of 46.5% and 67.6%, respectively [12, 24]. Hypoxia-induced hepatocellular injury impairs bilirubin uptake and excretion, further increasing serum bilirubin levels [25]. Collectively, these disturbances contribute to an elevated postoperative ALBI score and its association with adverse clinical outcomes.

The findings of the present study have notable clinical implications. The ALBI score is a simple, objective, and readily accessible metric derived from routine serum albumin and total bilirubin laboratory tests, which enables convenient clinical application. Stratification of patients at high postoperative risk based on the postALBI score may assist clinicians in formulating individualized perioperative management strategies. These strategies include optimized fluid resuscitation, targeted anti-inflammatory treatment, nutritional intervention to improve albumin status, and enhanced multi-organ function monitoring, which may contribute to improved postoperative outcomes in ATAAD patients. Furthermore, the postALBI score has the potential to serve as a practical surrogate endpoint for future clinical trials evaluating novel therapeutic strategies for this high-risk patient cohort.

Several limitations of this study should be acknowledged. First, this was a single-center retrospective study, which may carry inherent selection bias and limit the external generalizability of the results. Although PSM was applied to balance baseline characteristics, residual confounding cannot be entirely excluded. Second, the relatively small sample size may reduce the statistical power of certain analyses. Third, the precise mechanisms linking the postoperative ALBI score to adverse outcomes were not directly explored, and further experimental and clinical studies are needed to clarify these pathways. Finally, the optimal cutoff value of the ALBI score for risk stratification in ATAAD patients requires validation in large-scale multicenter studies.

In conclusion, the postALBI score represents a reliable, convenient, and independent prognostic marker for multiple adverse clinical outcomes in ATAAD patients undergoing emergency surgical repair. Routine implementation of this score in clinical practice may facilitate risk stratification, guide therapeutic decision-making, and improve perioperative management. Large-scale multicenter prospective studies are warranted to validate these findings and further define the clinical role of the ALBI score in this high-risk population.

## Supplementary Information


Supplementary Material 1.



Supplementary Material 2.



Supplementary Material 3.


## Data Availability

The raw data supporting the conclusions of this article will be made available by the authors on request.
