# Supplementary material for: Prognostic value of postoperative albumin-bilirubin score in patients with acute type A aortic dissection after surgery: a propensity-score matched analysis
Source: BMC Cardiovasc Disord. 2026 Jun 22;26:766. doi: 10.1186/s12872-026-06058-3 (PMC13536763; doi:10.1186/s12872-026-06058-3)
Supplement: Supplementary file 1 [file 12872_2026_6058_MOESM1_ESM.docx]

Supplemental Table Sensitivity analysis based on multiple imputation for missing data

| **Variables** | **MI_5** | **P value** | **MI_10** | **P value** | **CC** | **P value** |
| --- | --- | --- | --- | --- | --- | --- |
| **Delayed extubation** | 2.042[1.176, 3.546] | 0.011 | 2.950[1.522, 5.716] | 0.001 | 3.025[1.618, 5.654] | <0.001 |
| **Reintubation** | 3.949[1.712,9.109] | 0.001 | 2.087[0.948, 4.595] | 0.068 | 2.087[0.948, 4.595] | 0.068 |
| **Neurological dysfunction** | 1.590[0.738, 3.325 | 0.237 | 2.155[0.885, 5.429] | 0.091 | 2.155[0.885, 5.249] | 0.091 |
| **CRRT** | 4.352[2.162, 8.758] | <0.001 | 3.825[1.913, 7.649] | <0.001 | 3.825[1.913, 7.649] | <0.001 |
| **30-day mortality** | 1.898[0.976, 3.693] | 0.059 | 2.803[1.182, 6.647] | 0.019 | 2.803[1.182, 6.647] | 0.019 |

MI= multiple imputation; CC= Complete-case analysis; CRRT = continuous renal replacement therapy

Supplemental Figure. Love plot of standardized mean differences (SMD) for baseline covariates before and after propensity score matching.

Red dots represent SMD values before PSM, and blue dots represent SMD values after PSM. The vertical dashed lines indicate the balanced threshold of SMD = 0.1.
