# Supplementary material for: Prognostic value of postoperative albumin-bilirubin score in patients with acute type A aortic dissection after surgery: a propensity-score matched analysis
Source: BMC Cardiovasc Disord. 2026 Jun 22;26:766. doi: 10.1186/s12872-026-06058-3 (PMC13536763; doi:10.1186/s12872-026-06058-3)
Supplement: Supplementary file 2 [file 12872_2026_6058_MOESM2_ESM.docx]

| **Section/Topic** | **Item** | **Checklist Item** | **Page** |
| --- | --- | --- | --- |
| **Title and abstract** | | | |
| Title | 1 | Identify the study as developing and/or validating a multivariable prediction model, the target population, and the outcome to be predicted. | **1** |
| Abstract | 2 | Provide a summary of objectives, study design, setting, participants, sample size, predictors, outcome, statistical analysis, results, and conclusions. | **2** |
| **Introduction** | | | |
| Background and objectives | 3a | Explain the medical context (including whether diagnostic or prognostic) and rationale for developing or validating the multivariable prediction model, including references to existing models. | **4-5** |
|  | 3b | Specify the objectives, including whether the study describes the development or validation of the model or both.  The primary objective of this study was to explore the prognostic value of postALBI score in patients undergoing emergency surgery for acute type A aortic dissection. This study mainly focused on developing a prognostic prediction model to confirm that elevated postALBI score acts as an independent risk factor for multiple postoperative adverse events and 30-day mortality. Internal predictive performance of the postALBI score was assessed using ROC curve analysis in the enrolled cohort. No separate external validation cohort was established in the current research. | **5** |
| **Methods** | | | |
| Source of data | 4a | Describe the study design or source of data (e.g., randomized trial, cohort, or registry data), separately for the development and validation data sets, if applicable. | **5-6** |
|  | 4b | Specify the key study dates, including start of accrual; end of accrual; and, if applicable, end of follow-up. | **5-6** |
| Participants | 5a | Specify key elements of the study setting (e.g., primary care, secondary care, general population) including number and location of centres. | **5-6** |
|  | 5b | Describe eligibility criteria for participants. | **5** |
|  | 5c | Give details of treatments received, if relevant. | **NO** |
| Outcome | 6a | Clearly define the outcome that is predicted by the prediction model, including how and when assessed. | **6** |
|  | 6b | Report any actions to blind assessment of the outcome to be predicted. | **NO** |
| Predictors | 7a | Clearly define all predictors used in developing or validating the multivariable prediction model, including how and when they were measured. | **7** |
|  | 7b | Report any actions to blind assessment of predictors for the outcome and other predictors. | **NO** |
| Sample size | 8 | Explain how the study size was arrived at. | **5** |
| Missing data | 9 | Describe how missing data were handled (e.g., complete-case analysis, single imputation, multiple imputation) with details of any imputation method.  In this single-center retrospective study, all core clinical data, postoperative liver function indicators and follow-up outcome information were completely recorded in our hospital electronic medical system. No obvious missing key variables were observed in the enrolled participants. For a small number of occasional missing auxiliary laboratory indices, we adopted complete-case analysis by excluding individuals with incomplete data. No multiple imputation or other missing data filling methods were applied in this research. | NO |
| Statistical analysis methods | 10c | For validation, describe how the predictions were calculated.  The predictive performance was evaluated based on the postoperative ALBI score.  The ALBI score was calculated using the established formula:  ALBI = log₁₀(bilirubin (μmol/L)) × 0.66 − albumin (g/L) × 0.085  According to the optimal cutoff value (−2.12) derived from ROC curve analysis, patients were stratified into low and high postALBI groups.  The predictive probabilities for each adverse clinical outcome were estimated via multivariate logistic regression models, and the discriminatory ability was quantified by calculating AUC values. | **6-7** |
|  | 10d | Specify all measures used to assess model performance and, if relevant, to compare multiple models. | **7-8** |
|  | 10e | Describe any model updating (e.g., recalibration) arising from the validation, if done. | **NO** |
| Risk groups | 11 | Provide details on how risk groups were created, if done. | **7** |
| Development vs. validation | 12 | For validation, identify any differences from the development data in setting, eligibility criteria, outcome, and predictors.  In this study, all analyses were performed within the same single-center retrospective cohort. There was no separate external validation dataset. No differences existed between internal development data and validation data in terms of research setting, inclusion and exclusion criteria, definition of postoperative adverse outcomes, and included predictive variables. All study definitions and research conditions remained consistent throughout the whole analysis process. | **NO** |
| **Results** | | | |
| Participants | 13a | Describe the flow of participants through the study, including the number of participants with and without the outcome and, if applicable, a summary of the follow-up time. A diagram may be helpful. | **5** |
|  | 13b | Describe the characteristics of the participants (basic demographics, clinical features, available predictors), including the number of participants with missing data for predictors and outcome. | **5-7** |
|  | 13c | For validation, show a comparison with the development data of the distribution of important variables (demographics, predictors and outcome).  Since this study only adopted a single unified patient cohort without independent external validation data, all participants were used as both development data and internal verification data. The distributions of demographic characteristics, core predictive variable (postoperative ALBI score) and all defined postoperative adverse outcomes were completely consistent across the whole study population. After 1:1 propensity score matching, the distributions of all baseline demographic and clinical variables were well balanced between different risk groups, which further ensured consistent variable distribution and stable research results. | **8-10** |
| Model performance | 16 | Report performance measures (with CIs) for the prediction model. | **9-10** |
| Model-updating | 17 | If done, report the results from any model updating (i.e., model specification, model performance). | **NO** |
| **Discussion** | | | |
| Limitations | 18 | Discuss any limitations of the study (such as nonrepresentative sample, few events per predictor, missing data). | **13** |
| Interpretation | 19a | For validation, discuss the results with reference to performance in the development data, and any other validation data.  The present study was designed as a single-center retrospective observational study, and all enrolled patients were served as the original development dataset to establish the predictive effect of postALBI score. In this internal cohort, we evaluated the predictive performance via ROC curve analysis, and calculated corresponding AUC values to confirm satisfactory discriminatory power of postALBI score for various postoperative adverse outcomes and 30-day mortality. Given the limited research conditions, independent external validation cohort data were not available in this study. Further multi-center prospective studies with external validation are still required to verify the generalizability and universal applicability of our findings. | **NO** |
|  | 19b | Give an overall interpretation of the results, considering objectives, limitations, results from similar studies, and other relevant evidence. | **10-13** |
| Implications | 20 | Discuss the potential clinical use of the model and implications for future research. | **12-13** |
| **Other information** | | | |
| Supplementary information | 21 | Provide information about the availability of supplementary resources, such as study protocol, Web calculator, and data sets. | **NO** |
| Funding | 22 | Give the source of funding and the role of the funders for the present study. | **13** |

We recommend using the TRIPOD Checklist in conjunction with the TRIPOD Explanation and Elaboration document.
