# Supplementary material for: Prognostic value of postoperative albumin-bilirubin score in patients with acute type A aortic dissection after surgery: a propensity-score matched analysis
Source: BMC Cardiovasc Disord. 2026 Jun 22;26:766. doi: 10.1186/s12872-026-06058-3 (PMC13536763; doi:10.1186/s12872-026-06058-3)
Supplement: Supplementary file 3 [file 12872_2026_6058_MOESM3_ESM.doc]

STROBE Statement—Checklist of items that should be included in reports of ***cross-sectional studies***

|  | Item No | Recommendation | Pages |
| --- | --- | --- | --- |
| **Title and abstract** | 1 | (*a*) Indicate the study’s design with a commonly used term in the title or the abstract | 1-2 |
|  |  | (*b*) Provide in the abstract an informative and balanced summary of what was done and what was found | 2-3 |
| Introduction | | |  |
| Background/rationale | 2 | Explain the scientific background and rationale for the investigation being reported | 4-5 |
| Objectives | 3 | State specific objectives, including any prespecified hypotheses | 5 |
| Methods | | |  |
| Study design | 4 | Present key elements of study design early in the paper | 5 |
| Setting | 5 | Describe the setting, locations, and relevant dates, including periods of recruitment, exposure, follow-up, and data collection | 5-6 |
| Participants | 6 | (*a*) Give the eligibility criteria, and the sources and methods of selection of participants | 5-6 |
| Variables | 7 | Clearly define all outcomes, exposures, predictors, potential confounders, and effect modifiers. Give diagnostic criteria, if applicable | 6 |
| Data sources/ measurement | 8* | For each variable of interest, give sources of data and details of methods of assessment (measurement). Describe comparability of assessment methods if there is more than one group | 7 |
| Bias | 9 | Describe any efforts to address potential sources of bias | 7 |
| Study size | 10 | Explain how the study size was arrived at | 5 |
| Quantitative variables | 11 | Explain how quantitative variables were handled in the analyses. If applicable, describe which groupings were chosen and why | 7 |
| Statistical methods | 12 | (*a*) Describe all statistical methods, including those used to control for confounding | 7 |
|  |  | (*b*) Describe any methods used to examine subgroups and interactions  Subgroup analyses were not formally performed in the present study. To minimize potential subgroup-derived confounding effects between groups, we conducted 1:1 propensity score matching to balance all baseline demographic and clinical covariates including age, gender, BMI, hypertension history and smoking status. No formal interaction term analysis was conducted in this study. All clinically relevant confounding variables were incorporated into multivariate logistic regression models to adjust for mutual confounding effects and ensure the stable independent predictive effect of postoperative ALBI score on adverse postoperative outcomes. | NO |
|  |  | (*c*) Explain how missing data were addressed  In this single-center retrospective study, all core clinical data, postoperative liver function indicators and follow-up outcome information were completely recorded in our hospital electronic medical system. No obvious missing key variables were observed in the enrolled participants. For a small number of occasional missing auxiliary laboratory indices, we adopted complete-case analysis by excluding individuals with incomplete data. No multiple imputation or other missing data filling methods were applied in this research. | NO |
|  |  | (*d*) If applicable, describe analytical methods taking account of sampling strategy  This study adopted a consecutive sampling strategy. All eligible patients with acute type A aortic dissection who underwent emergency surgery during the study period were consecutively enrolled without random sampling. Given the non-randomized consecutive enrollment design, we performed 1:1 propensity score matching to balance baseline confounding factors between different postALBI groups, so as to reduce selection bias and make the inter-group comparison results more reliable. | NO |
|  |  | (*e*) Describe any sensitivity analyses  No additional sensitivity analyses were conducted. The stability of results was guaranteed by strict enrollment criteria, propensity score matching-based baseline balance and consistent confounder adjustment in regression models. | NO |
| Results | | |  |
| Participants | 13* | (a) Report numbers of individuals at each stage of study—eg numbers potentially eligible, examined for eligibility, confirmed eligible, included in the study, completing follow-up, and analysed | 5-6 |
|  |  | (b) Give reasons for non-participation at each stage | 5-6 |
|  |  | (c) Consider use of a flow diagram | 5-6 |
| Descriptive data | 14* | (a) Give characteristics of study participants (eg demographic, clinical, social) and information on exposures and potential confounders | 8 |
|  |  | (b) Indicate number of participants with missing data for each variable of interest | 5 |
| Outcome data | 15* | Report numbers of outcome events or summary measures | 8 |
| Main results | 16 | (*a*) Give unadjusted estimates and, if applicable, confounder-adjusted estimates and their precision (eg, 95% confidence interval). Make clear which confounders were adjusted for and why they were included | 9 |
|  |  | (*b*) Report category boundaries when continuous variables were categorized | 7 |
|  |  | (*c*) If relevant, consider translating estimates of relative risk into absolute risk for a meaningful time period  In this study, we calculated and reported both relative risk indicators (odds ratios) and absolute event rates within the unified follow-up period. All adverse clinical outcomes were observed within 30 days after emergency surgery. We presented the raw incidence rates of delayed extubation, CRRT use, neurological dysfunction, reintubation and 30-day mortality in low and high postALBI groups respectively, which directly reflected the absolute postoperative risk differences between the two groups during this critical observation window. |  |
| Other analyses | 17 | Report other analyses done—eg analyses of subgroups and interactions, and sensitivity analyses | 7-8 |
| Discussion | | |  |
| Key results | 18 | Summarise key results with reference to study objectives | 12-13 |
| Limitations | 19 | Discuss limitations of the study, taking into account sources of potential bias or imprecision. Discuss both direction and magnitude of any potential bias | 12 |
| Interpretation | 20 | Give a cautious overall interpretation of results considering objectives, limitations, multiplicity of analyses, results from similar studies, and other relevant evidence | 10-12 |
| Generalisability | 21 | Discuss the generalisability (external validity) of the study results | 12 |
| Other information | | |  |
| Funding | 22 | Give the source of funding and the role of the funders for the present study and, if applicable, for the original study on which the present article is based | 13 |

*Give information separately for exposed and unexposed groups.

**Note:** An Explanation and Elaboration article discusses each checklist item and gives methodological background and published examples of transparent reporting. The STROBE checklist is best used in conjunction with this article (freely available on the Web sites of PLoS Medicine at http://www.plosmedicine.org/, Annals of Internal Medicine at http://www.annals.org/, and Epidemiology at http://www.epidem.com/). Information on the STROBE Initiative is available at www.strobe-statement.org.
